# Diffuse PRAME Expression in Transdifferentiated Melanomas

**DOI:** 10.1111/cup.14769

**Published:** 2024-12-03

**Authors:** Shaymaa Hegazy, Arivarasan Karunamurthy, Ivy John

**Affiliations:** ^1^ Department of Pathology University of Pittsburgh Medical Center Pittsburgh Pennsylvania USA

Dedifferentiated melanoma is defined by the absence of typical histopathologic and immunophenotypic features associated with melanoma. Much like dedifferentiated liposarcomas and malignant peripheral nerve sheath tumors, dedifferentiated melanomas exhibit a propensity for heterologous transdifferentiation, showcasing a spectrum of phenotypes including rhabdomyosarcomatous‐like, fibroblastic/myofibroblastic‐like, adenocarcinoma‐like, leiomyosarcoma‐like, schwannian and perineural‐like, teratocarcinosarcoma‐like, and chondrosarcomatous‐like [1, 2]. Accurate diagnosis of dedifferentiated melanoma demands a high level of suspicion and comprehensive sampling to identify either a conventional melanoma precursor or mutations consistent with melanoma, such as *BRAF, NRAS, or NF1* [1, 3]. In recent years, immunohistochemistry focusing on PRAME (preferentially expressed in melanoma) has emerged as a valuable diagnostic tool for melanocytic tumors. The vast majority of primary and metastatic cutaneous melanomas express PRAME, though exceptions may arise, particularly in desmoplastic melanomas [4]. Rare, isolated case reports and a recent single study have reported strong and diffuse PRAME expression in dedifferentiated melanomas; however, its expression in transdifferentiated melanomas remains underexplored [5, 6, 7, 8]. Herein, we present the findings of PRAME expression in three cases of transdifferentiated melanomas.

## 1 Case 1

**FIGURE 1:**
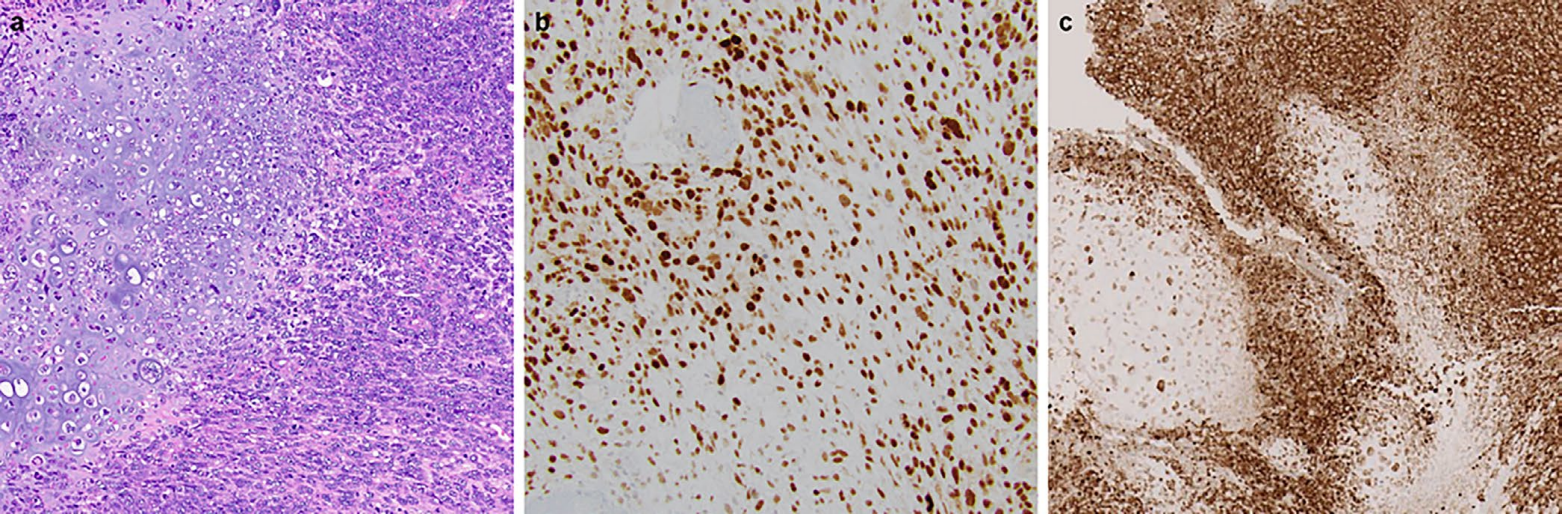
Dedifferentiated melanoma with chondroblastic differentiation (a, H&E, 100×), showing diffuse and strong PRAME expression (b, 200×). BRAF immunohistochemistry shows positivity in the tumor cells (c, 100×).

Received in consultation is a 15‐cm groin mass with an outside diagnosis of high‐grade malignant neoplasm with chondrosarcomatous differentiation with a suggested differential diagnosis of dedifferentiated chondrosarcoma and chondroblastic osteosarcoma. The submitted sections showed morphological features reminiscent of dedifferentiated chondrosarcoma with an abrupt transition between conventional hyaline cartilage and malignant sarcomatoid neoplasm (Figure 1a). Further history revealed the patient's documented *BRAF V600E* mutated malignant melanoma originating from the left thigh, with biopsy‐proven lymph node metastasis to the neck. Although conventional melanocytic markers were negative in the current specimen, immunohistochemical staining for PRAME revealed diffuse and strong nuclear expression in the sarcomatoid component (Figure 1b). Additionally, BRAF immunohistochemistry confirmed positivity in the tumor cells (Figure 1c). Molecular studies were performed comparing the current specimen (left groin mass) and metastatic melanoma to the neck and showed identical mutations involving *BRAF, TERT, and TP53* in both components, confirming that both tumors are clonally related. Therefore, a diagnosis of dedifferentiated melanoma with chondrosarcomatous differentiation was rendered.

## 2 Case 2

**FIGURE 2:**
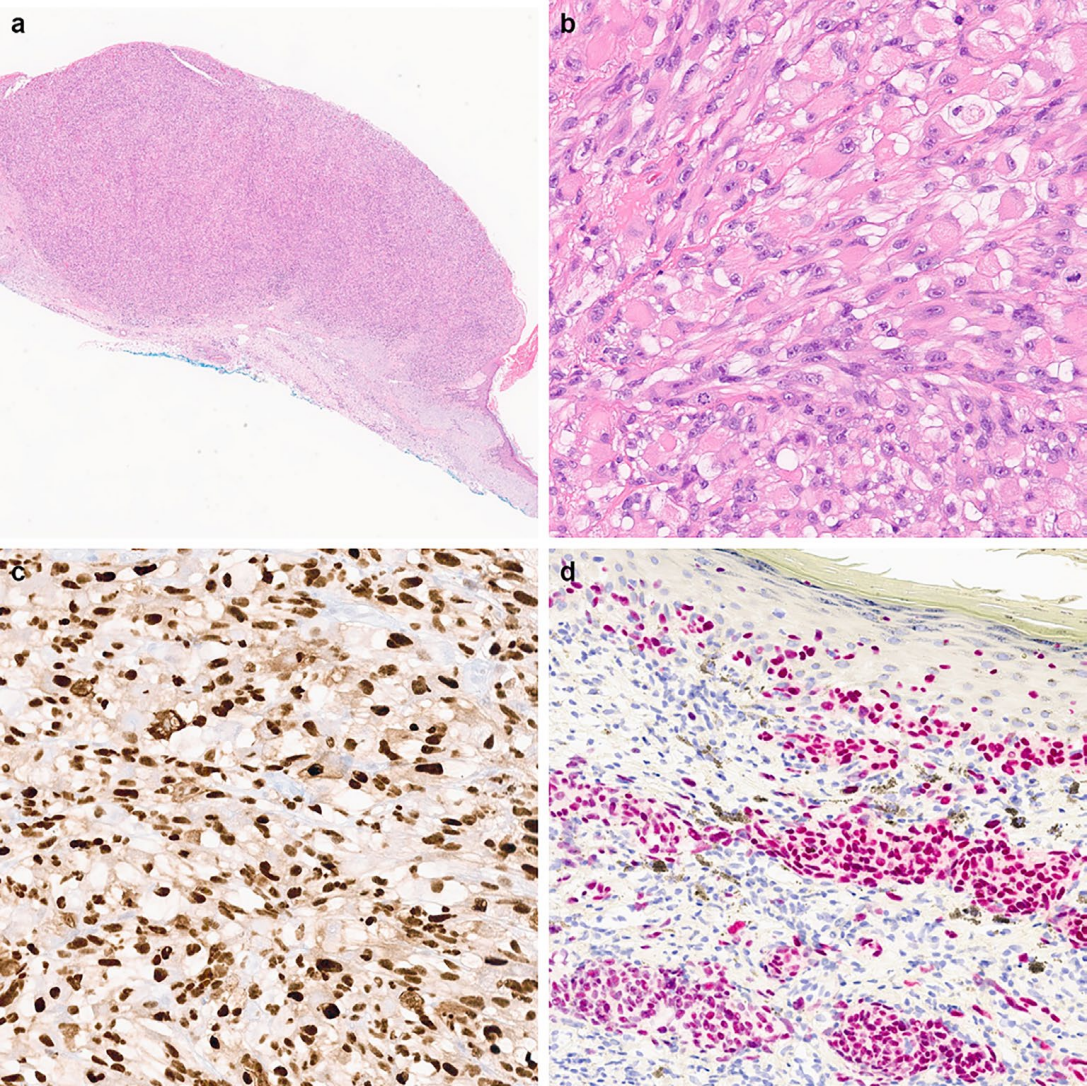
Dedifferentiated melanoma with rhabdomyoblastic differentiation (a, H&E, 10×) and (b, 200×). The tumor cells show diffuse and strong PRAME expression (c, 200×) and multifocal positivity with SOX10 (d, 200×).

As part of ongoing patient care, histologic slides from a cutaneous rhabdomyosarcoma involving the forearm were received for review. Histopathologic examination showed a well‐circumscribed polypoid dermal‐based neoplasm with an epidermal collarette composed of rhabdoid to spindle cells arranged in sheets and fascicles (Figure 2a,b). In addition, ulceration of the overlying skin, significant background solar elastosis, and peripheral lymphoid aggregates were noted. The accompanying desmin and myogenin stains were positive, confirming rhabdomyoblastic differentiation. Melanocytic markers were performed, including SOX10, S100, Melan A, HMB45, and PRAME, and showed diffuse PRAME expression, while other stains were negative (Figure 2c). Subsequent repeat SOX10, S100, Melan A, and HMB45 on additional blocks revealed multifocal positivity with SOX10 on a single block (Figure 2d). Therefore, a diagnosis of dedifferentiated melanoma with rhabdomyoblastic differentiation was rendered.

## 3 Case 3

A core needle biopsy of an axillary mass in a patient with a history of nodular melanoma of the scalp revealed a cellular proliferation of rhabdoid cells characterized by abundant eosinophilic cytoplasm and eccentrically placed nuclei. Immunohistochemical staining confirmed rhabdomyoblastic differentiation, as the tumor cells tested positive for desmin and myogenin. Additionally, PRAME exhibited diffuse and strong positivity, while other conventional melanocytic markers yielded negative results. Further analysis through targeted next‐generation sequencing identified several genomic alterations, including a *TERT* mutation (C250T), an *NRAS* mutation (p.Q61L), and an *NF1* mutation. The tumor demonstrated microsatellite stability and a tumor mutation burden of 26 mutations per megabase. In the context of the patient's history, these genetic alterations strongly support a diagnosis of metastatic dedifferentiated melanoma with rhabdomyoblastic differentiation.

These cases confirm the diffuse PRAME expression in dedifferentiated melanomas, including transdifferentiated subsets. Notably, while previous documentation exists of PRAME expression in a few dedifferentiated melanomas with rhabdomyoblastic differentiation, this study marks the first report of its expression in cases with chondrosarcomatous differentiation [7, 8]. It is crucial to acknowledge that PRAME staining is not exclusive to melanocytic neoplasms and has been observed in various non‐melanocytic tumors as well, albeit often variable [6, 8]. However, it remains a valuable initial screening tool in diagnostically challenging cases. The observation of diffuse PRAME expression should prompt further molecular analysis to explore potential underlying melanoma‐compatible mutations, particularly in appropriate clinical settings.

## Conflicts of Interest

The authors declare no conflicts of interest.

## Data Availability

Data sharing is not applicable to this article as no new data were created or analyzed in this study.
